# Focusing on antimicrobial resistant infections –are we missing the forest for the trees and the patients for pathogens?

**DOI:** 10.3389/frabi.2023.1329081

**Published:** 2023-12-19

**Authors:** Alexander Lawandi, Sameer S. Kadri, John H. Powers

**Affiliations:** ^1^ Critical Care Medicine Department, National Institutes of Health Clinical Center, Bethesda, MD, United States; ^2^ Division of Infectious Diseases, Department of Medicine, McGill University Health Center, Montreal, QC, Canada; ^3^ Division of Medical Microbiology, Department of Laboratory Medicine, McGill University Health Centre, Montreal, QC, Canada; ^4^ Department of Critical Care Medicine, McGill University Health Centre, Montreal, QC, Canada; ^5^ Critical Care Medicine Branch, National Heart, Lung, Blood Institute, Bethesda, MD, United States; ^6^ Clinical Research Directorate, Frederick National Laboratory for Cancer Research, Frederick, MD, United States

**Keywords:** antimicrobial resistance, bacterial infections, patient outcomes, clinical trial design, infectious diseases

## Abstract

Antimicrobial resistance (AMR) is a challenge because it is associated with worse patient outcomes. To solve the problem will take development of interventions and policies which improve patient outcomes by prolonging survival, improving patient symptoms, function and quality of life. Logically, we should look to focusing resources in areas that would have the greatest impact on public health. AMR takes the approach of focusing on individual pathogens and “pathogen-focused” development. However, evaluating overall infections and their impact on patient outcomes reveals that 17 of 18 infection deaths are associated with susceptible pathogens. Here we discuss recentering on patients and patient outcomes instead of pathogens, and propose six suggestions on how a patient focus impacts areas and incentives for clinical research.

## Introduction

Antimicrobial resistance (AMR) has been prioritized as a global health threat due to its impact on patient outcomes. A recent report from the Antimicrobial Resistance Collaborators (Murray et al., 2022) estimated that nearly five million deaths occurred globally in 2019 in association with drug resistant pathogens, with approximately 1.27 million estimated directly attributable deaths. While these estimates are undoubtedly concerning, there are two important *caveats* to bear in mind. Firstly, most studies on AMR burden provide numerator estimates that solely focus on disease caused by resistant organisms, taking those numbers out of their larger context. The denominator of overall infection-related deaths is often unknown, unreported, or de-emphasized, and in many parts of the world, is several-fold larger. Secondly, not all *in vitro* resistance is equal when it comes to patient outcomes. Yet, burden estimates often conflate treatment-limiting resistance phenotypes, such as extensively/pan drug resistance or difficult to treat resistance phenotype (DTR, meaning where all first line, highly safe and efficacious antibiotics would be considered ineffective), with those that remain treatable with currently available drugs, such as resistance to only one antibiotic e.g. a fluoroquinolone (FQR). Treating these scenarios as equivalent for patients can skew reported AMR burdens, impressions and downstream action.

What’s the alternative? Would we reconfigure our interpretation of the mortality estimates if we consider the broader context of all infection-related deaths regardless of susceptible or resistant pathogens? Would a more granular analysis of the resistance phenotypes associated with these deaths change our prioritization of resistant pathogens to all bacterial pathogens regardless of the *in vitro* susceptibility results? Would the broader context point the way to the kinds of patients most in need, how to identify them, and to developing different interventions needed to improve patient outcomes?

A recent report estimating the burden of AMR mortality in Europe addresses the first of the caveats (Mestrovic et al., 2022). Using the same methodology as the Antimicrobial Resistance Collaborators, researchers identified that 1.2 million deaths in Europe in 2019 were associated with infection in general, and not simply associated with AMR. Of these, just under 60% were associated with pathogens displaying any kind of resistance pattern, even resistance to less commonly used single agents like aminoglycosides. Their overall infection-associated death toll estimate of 1.2 million deaths in Europe provides a sobering reminder that disease due to resistant bacteria is just one part of a larger problem. Irrespective of resistance, there is a considerable mortality from bacterial infections overall. If one were to apply a more clinically useful patient-centered definition of resistance to only capture disease from bacteria with limitations in clinical therapy, for example the Difficult to Treat (DTR) phenotype, then one would reveal most infected-related deaths in Europe due to susceptible pathogens, as posed by several investigators (Abat et al., 2017; Raoult et al., 2019; Diallo et al., 2020).

Recent work in the US has allowed for a clear understanding of the impact of restricting analyses of the burden of AMR to deaths to clinically relevant resistance. An analysis of over 50,000 U.S. patients with gram-negative bloodstream infections (BSIs) showed approximately 1% display the DTR phenotype (Kadri et al., 2018). Putting this number in context reveals the converse: ~99% of patients had at least one “good” agent with *in vitro* activity available for treatment. In this study, among patients with DTR bloodstream pathogens there was a high mortality rate of 43%, likely in part from inadequate empiric therapy, highly toxic and/or sub efficacious targeted therapy, and disordered host-immune responses to infection irrespective of *in vitro* susceptibility. In comparison, although mortality rates were lower at ~15% in patients with susceptible gram-negative infections, these cases were considerably more common with higher overall burden of disease, leading to 3,161 deaths in highly susceptible disease vs 190 deaths associated with DTR (a ratio 17 deaths with susceptible pathogens for every 1 death with a DTR pathogen). Such resistance metrics with prognostic utility that might exert similarly limiting effects on patient management in different global regions might yield useful geographic comparisons of resistance burdens.

Unfortunately, the prioritization of “bugs and drugs” over patient outcomes seems to have moved susceptible infections into a stakeholder blind spot. It is critical yet underappreciated that “effective” antibiotic therapies with *in vitro* biological activity do not guarantee patient survival. The considerable investment into the development of antibiotics targeting resistant pathogens is important but will only improve the overall outcomes of patients to a certain point. Without concerted efforts to also counter avoidable deaths in patients with susceptible disease by clinical and public health measures including prevention, earlier recognition, better diagnostics, prompt treatment and exploring better host directed therapies, metrics on infection-related mortality are unlikely to improve greatly. The single focus by both governmental and non-governmental organizations exclusively on AMR may inadvertently mislead clinicians, patients, and other stakeholders to believe that susceptible infections pose minimal threat and consequently, lead to their neglect in terms of study and funding of this entity that would have a greater impact on decreasing overall infection related burden for patients. Careful consideration of the epidemiology of bacterial infections supports a need for better interventions that improve patient outcomes, if not more so, for disease due to pathogens for which treatment options with *in vitro* activity are readily available but in whom the burden of disease and poor outcomes are still substantial.

## Discussion

Here we pose six suggestions to help improve patient outcomes in bacterial infections and refocus incentives (**Table 1**).

**Table 1:** Refocusing on patients rather than pathogens: Implications for research and incentives.

Surveillance on patients and outcomes in both antibiotic susceptible and resistant disease focusing on baseline patient and laboratory risk factors for poor patient outcomes
Development of rapid point of care diagnostics with demonstrated evidence of improving patient outcomes to better focus administration and proper use of new and older agents
Patient enrollment in trials based on lack of effective interventions regardless of causative pathogen to increase feasibility and relevance of clinical trials
Interventions beyond small molecule antimicrobials including host directed therapies (including vaccines), microbiome, phage and other types of interventions
Shift from non-inferiority trial hypotheses to superiority to improve outcomes for patients when current standards of care are not offering acceptable outcomes
Outcomes based on direct measures of patient’s health including survival, patient symptoms and function in their daily lives measured by valid Patient Reported Outcomes

### Patient outcomes: tracking patient outcomes regardless of pathogens

First, surveillance and burden estimates should focus on patient outcomes and patient factors that affect outcomes rather than just tracking organism resistance patterns *in vitro*. This would allow a better assessment of what types of patients experience lack of response to available therapies regardless of pathogen susceptibility. There is little research defining “not getting better” while receiving current therapies in common infections other than risk factors for mortality. Well-designed observational studies could evaluate which patient and laboratory baseline risk factors are prognostic for worse outcomes on current therapies across a range of parameters (for instance length of stay, increased severity of illness at presentation, need for higher levels of care etc.) and define enrollment criteria for future studies. Recognizing which patients are at risk for decompensation, who may need higher levels of care and intervention, may improve outcomes for all patients, and not simply those with resistant pathogens (Adams et al., 2022).

### Better diagnostics that improve patient outcomes

Second, better diagnostics are needed to apply current as well as new therapies. A large US study showed one in every five inpatients with BSI received inappropriate empiric antibiotic therapy (Kadri et al., 2021). Half of instances of “inappropriate” empiric therapy were with susceptible pathogens. Such empiricism is due to lack of rapid point of care diagnostics to aid accurate prescribing decisions. Enhanced investment in novel applications of rapid phenotypic tests could provide shorter times to appropriate therapy (Del Corpo et al., 2023) and improve patient outcomes. This is particularly relevant for areas with lower rates of resistance in which the inappropriate overuse of broad-spectrum antibiotics has been associated with worse outcomes (Rhee et al., 2020).

### Patients: focus on those in whom current therapies are not effective regardless of pathogen

Third, expanding the focus from patients with AMR to include all patients who lack effective options, or in whom current therapies fail despite appropriate therapy regardless of pathogens, would expand the focus of clinical research, improving enrollment and clinical relevance of trials. Strich et al., using a combination of pharmacological and microbiological data, characterized the number of treatment opportunities for novel antimicrobials in U.S. hospitals. The authors identified the niche for novel antimicrobials as conspicuously small, especially relative to the 70-fold larger volume of treatment opportunities identified for therapies targeting patients with susceptible infections (Strich et al., 2020). Yet, there is still discussion of “pathogen-focused development” rather than patient-focused development despite current trials enrolling few patients with resistant pathogens to available drugs (Yahav et al., 2021b). The types of interventions evaluated needs to expand beyond small molecule drugs that inhibit organism growth. Greater focus is needed on the development of vaccines to prevent the acquisition of infection in the first place. Fortunately, vaccines targeting *Klebsiella pneumoniae* (Assoni et al., 2021), *Pseudomonas aeruginosa* (Merakou et al., 2018), and *Staphylococcus aureus* (Moscoso et al., 2018), three pathogens associated with significant morbidity and mortality, are under study and hopefully will bear fruit comparable to the dramatic reductions in pediatric deaths associated with *Streptococcus pneumoniae* and *Haemophilus influenzae* vaccination (Wahl et al., 2018). Such interventions would benefit those with both susceptible and resistant disease. Similarly, the pathophysiology of sepsis revolves around a dysregulated host response to infection. From that perspective, new antibiotics may have little added benefit for many patients with sepsis. Therefore, study of host immunomodulators for patients with sepsis and septic shock will be key to improving their outcomes (Hutchins et al., 2014). An important driver of AMR rates is the use of antimicrobials themselves and therefore developing non-antimicrobial interventions such as microbiome or bacteriophage therapies may help limit the spread of AMR. Instead of claims of a return to a “pre-antibiotic era”, what is needed is a new “antibiotics-plus era” where antimicrobials as well as other types of interventions are available to improve all patient outcomes.

### Comparisons: evidence showing new interventions are superior in efficacy over current standards of care

Fourth, the questions posed by future studies should focus on superiority comparisons with current standards of care evaluating whether new interventions can improve patient outcomes, rather than questionable assumptions that *in vitro* activity of small molecule drugs studied in trials with non-inferiority hypotheses might benefit “future” unstudied types of patients. Non-inferiority trials are ethical and valid when evaluating added non-efficacy benefits like decreased adverse effects. But patients in whom current therapies are not effective need interventions studied with superiority hypotheses to evaluate if interventions have improved efficacy for them compared to current standards of care. The Belmont Report on research ethics points out there must be hypothesized benefits to balance harms for research participants in a given study (United S, 1978). A study cannot be justified by assumed benefits for future, unstudied types of patients. Yet the majority of current non-inferiority studies of new antibiotics enroll patients who already have effective therapies, exclude those who need better therapies (Kuzucan et al., 2020) and pose no hypotheses of benefits for the patients enrolled (Doshi et al., 2017). Despite the focus on resistant organisms, the majority of those enrolled have disease due to susceptible pathogens (Yahav et al., 2021b). The risk to patients from the focus on pathogens instead of patients is shown by the evidence with cefiderocol which demonstrated non-inferiority in patients with complicated urinary tract infections. The non-inferiority study allowed up to 20% less effectiveness in patients who already had effective therapies with no hypothesis regarding non-efficacy benefits (Portsmouth et al., 2018). A randomized “descriptive” trial with no hypothesis comparing cefiderocol to best available therapy in patients with a variety of diseases due to resistant pathogens against which cefiderocol had *in vitro* activity showed increased mortality with cefiderocol (Bassetti et al., 2021). These two studies exemplify how current NI studies do not enroll patients in whom current therapies are not effective regardless of *in vitro* susceptibility of the infecting organism (showing why it is challenging to demonstrate superiority) and that benefits cannot be assumed in sicker patients based on *in vitro* or animal data alone (Powers, 2021). Careful trial site selection in global regions with high burden of resistant organisms and adequate trial oversight might enable enrolling sufficient participants needed for superiority hypotheses. Expanding the types of interventions and enrolling patients in whom current drugs lack effectiveness regardless of *in vitro* susceptibility would further enable superiority trials.

### Outcomes: direct measures of patient outcomes

Fifth, outcomes especially in acute diseases should focus on direct measures of patient benefit such as improved survival, patient symptoms and patient function in their daily lives measured by valid Patient-Reported Outcome (PRO) tools. Recent analyses show clinical trials in severe infectious diseases may have non-patient centered outcomes such as changes in biomarkers or negative culture testing, as well as clinician reported outcomes which are indirect measures of patient outcomes. The validity of these outcomes in reflecting direct patient outcomes remain unclear (Timsit et al., 2017). The recommendations for trial designs by regulators routinely include surrogate endpoints, many of which have uncertain validity in evaluating benefits on patient outcomes. Future trials should restrict the outcomes for patients with infections, including resistant ones, to minimize endpoints of dubious clinical relevance and employ patient centered outcomes (Hey et al., 2020).

### Interventions: going beyond antibiotics

Developing appropriate incentives to solve a problem first requires correctly identifying the problem and then developing and testing valid solutions. As epidemiological evidence identifies susceptible disease as the greater health threat to patients in the US and possibly Europe, it seems like missing the forest for the trees to focus solely on small molecule antimicrobials and *in vitro* resistant pathogens instead of patients and patient outcomes. The U.S. government’s response has been significant but focused mainly on pathogens and resistance. The CDC has allotted nearly 500 million dollars to a range of programs to focusing on combat resistance at both the state and national levels (The Centers for Disease Control, 2023), the National Institutes of Health (NIH) has earmarked nearly 110 million dollars to fund research programs targeting antimicrobial resistance through the Antibiotic Resistance Leadership Group (National Institutes of Health, 2019), and the Biomedical Advanced Research and Development Authority (BARDA) has provided over 1.6 billion dollars to support the development of novel antimicrobials (Biomedical Advanced Research and Development Authority, 2021). While successful in bringing a greater quantity of drugs to market, the agents mostly have been modifications of existing drug classes. None of them have provided demonstrated evidence of improved direct patient outcomes and some have evidence of patient harm at increased cost (Deak et al., 2016; Yahav et al., 2021a; Mitra-Majumdar et al., 2022), leading to concern over the future of the antibiotic pipeline and a renewed call to action (Talbot et al., 2019). Since current incentives have no requirement for improved patient outcomes it is not surprising that evidence of added patient benefit is lacking, and these drugs have fared poorly in a functioning marketplace that declines to pay for drugs that do not demonstrate added patient benefits. Any incentives should focus on developing the evidence of demonstrated, rather than hypothetical, improved patient outcomes. Such interventions would benefit patients with both susceptible and resistant disease. The market size of those interventions would be larger and reimbursement would be based on value to patients in improving their outcomes.

## Conclusion

These suggestions are a starting point for refocusing development of medical interventions to provide value for patients and the health care system overall. Refocusing on patients instead of pathogens would provide added benefit to more patients, provide evidence of improved outcomes in patients with both susceptible and resistant disease, improve the feasibility and clinical relevance of clinical trials, and justify the cost of new interventions in a wider market. Discussions of the subset of AMR patients are important but should also include the larger group of patients with susceptible infections who need better interventions to improve outcomes.

## Data availability statement

The original contributions presented in the study are included in the article/supplementary material. Further inquiries can be directed to the corresponding author.

## Ethics statement

Ethical approval was not required for the study involving humans in accordance with the local legislation and institutional requirements. Written informed consent to participate in this study was not required from the participants or the participants’ legal guardians/next of kin in accordance with the national legislation and the institutional requirements.

## Author contributions

AL: Conceptualization, Data curation, Investigation, Writing – original draft, Writing – review & editing. SK: Conceptualization, Data curation, Formal analysis, Funding acquisition, Investigation, Methodology, Project administration, Resources, Supervision, Writing – original draft, Writing – review & editing. JP: Conceptualization, Data curation, Investigation, Methodology, Project administration, Resources, Writing – original draft.
